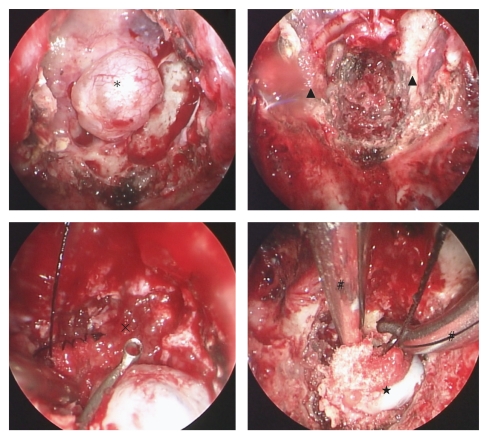# Technical Notes on Endoscopic Transnasal Transsphenoidal Approach for Clival Chondrosarcoma

**DOI:** 10.1155/2011/953047

**Published:** 2011-02-16

**Authors:** Atsushi Kuge, Shinya Sato, Kaori Sakurada, Sunao Takemura, Zensho Kikuchi, Yuki Saito, Takamasa Kayama

**Affiliations:** ^1^Department of Neurosurgery, Yamagata University Faculty of Medicine, 2-2-2 Iidanishi, Yamagata 990-9585, Japan; ^2^National Cancer Center, 5-1-1 Tsukiji, Chuo-ku, Tokyo 104-0045, Japan

## Abstract

Although there are various operative approaches for clival tumors, a transsphenoidal approach is one of choices when the main tumor extention is in an anterior-posterior direction with a slight lateral extension. However, this approach sometimes provides only narrow and deep operative field. Recently, endoscopic transnasal transsphenoidal approach is quite an effective approach for clival tumors because of the improvement of surgical instruments, image guidance systems, and techniques and materials of wound closure. In this paper, we describe the effectiveness, technical problems, and solution of this approach based on our experiences with two clival chondrosarcomas that was removed by endoscopic transnasal transsphenoidal approach.

## 1. Introduction

 Clival chondrosarcomas are rare group that are thought to originate from primitive mesenchymal cells or from the embryonal portion of the cartilaginous matrix of the cranium. And these tumors have aggressive features, infiltrative neoplasms. Although it is generally agreed that surgery is the choice of treatment for clival chondromas and chodrosarcomas, it is still controvertial whether it is preferable to attempt a radical resection, which implies wide exposure, extensive dissection, and the potential for significant surgical morbidity, or to determine the oncological features of the tumors and plan local control through more limited approaches. Transbasal, transmaxillary, transoral, transfacial, and subtemporal approaches have been used for clival lesion [1–5].

 On the other hand, transsphenoidal approach [6, 7] was used from 1960s [8], this approach was not widely spread because of narrow and deep operative field using microscope, and difficulty of wound closure complicated cerebrospinal fluid leakage [7]. However, an endoscopic transsphenoidal approach is now quite an effective approach for clival tumors because of the improvement of surgical instruments, image guidance systems, and materials of wound closure such as fibrin glue. In this paper, we describe the effectiveness, technical problems and solution of this approach based on our experiences with two clival chondrosarcomas that was removed by endoscopic transnasal transsphenoidal approach.

## 2. Patients and Methods

Consecutive two patients with a clival chondrosarcoma treated using an endonasal endoscopic approach were identified [9]. In all cases, frameless neurosurgical navigation (VectorVision: Brain LAB Co., Ltd.) was used.

**Case 1**

**Figure 1:**
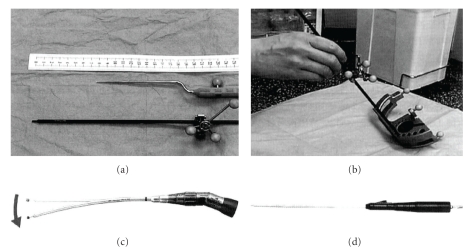
An original long navigation pointer (a) registered by the Universal Instrument Integration system ((b): VectorVision: BrainLAB Co., Ltd.). Long and slim instruments we used for this operation, highspeed drill ((c): Primado: Nakanishi Co., Ltd.) and ultrasonic aspirator ((d): SONOPET: M&M Co., Ltd.).

**Figure 2:**
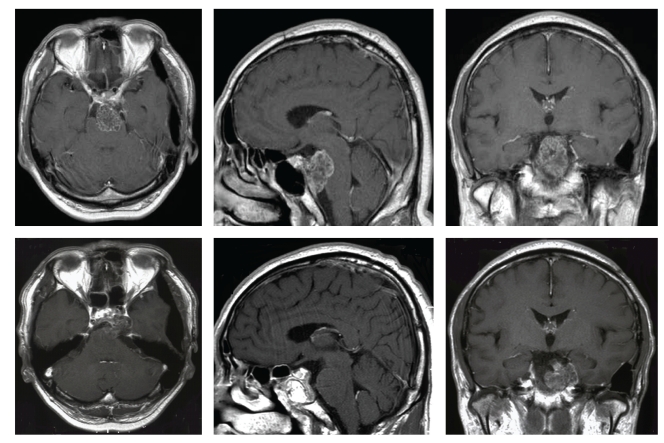
Case 1. Upper: MRI images 33 months after the first operation. The residual tumor is regrowing. Lower: MRI images after surgery, midline part of tumor was resected and decompressed brainstem.

A fifty-six-year-old male was suffering from diplopia. He showed left abducens nerve palsy, and MRI revealed clival enhanced mass lesion extending toward brainstem and cavernous sinus. First surgery was performed by craniotomy, and tumor was resected partially and get decompression of brainstem. Immunohistochemically, vimentin was positive, epithelial membrane antigen (EMA) and cytokeratin (CAM5.2) were negative, and pathological diagnosis was chondrosarcoma. Thirty-three months after first surgery, tumor enlargement was confirmed. Second surgery was performed by endoscopic transnasal transsphenoidal approach because tumor was mainly extended toward the posterior part. We used an endoscope with long axis (180 mm) and angled tip (0, 30, 70 degrees: HOPKINSII: KARL STORZ Co., Ltd.). And we made special navigation probe to get more correct information. It was registered by Universal Instrument Integration System (VectorVision: BrainLAB CO., Ltd.) which could make favorite length of navigation probe using materials you like. And instruments which long and slim curettes (Zonne Co., Ltd.), ultrasonic aspirator probe developed for abdominal surgery (SONOPET: M&M Co., Ltd.), high-speed drill (Primado: Nakanishi Co., Ltd.) which have curved handle did not to disturb operative field without the interference of each tools (Figure 1). We could get resection of main part of tumor without cavernous lesions. Dural defect was observed, and it was repaired by filling sphenoid sinus by autologous fat and fibrin glue. After surgery, new neurological deficit and cerebrospinal fluid leakage were not seen. Residual tumor regrowth was not observed for fifty months after surgery (Figure 2).

**Case 2**

**Figure 3:**
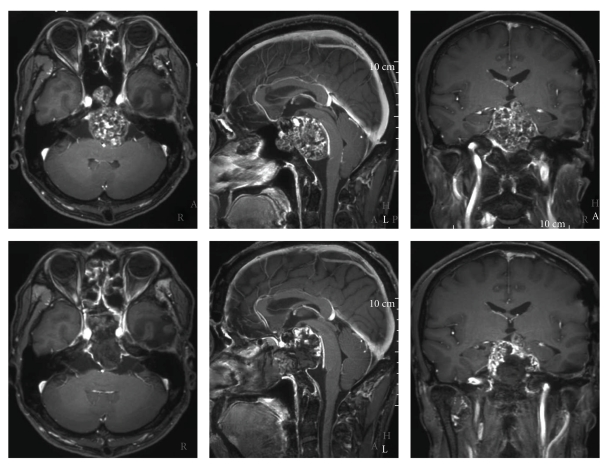
Case 2. Upper: MRI images after the second operation. Lower: MRI after transsphenoidal surgery. Tumor was resected except suprasellar part and decompressed brainstem.

**Figure 4:**
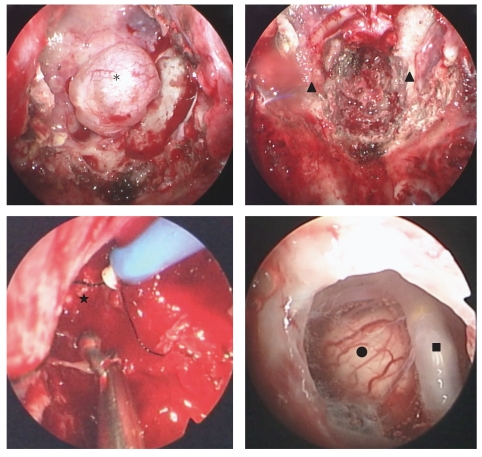
Case 2. Intraoperative endoscopic view. Upper left: tumor capsule extending sphenoid sinus (asterisk), right: bilateral carotid prominence and expose carotid artery (triangle), lower left: lateral part of the lesion (star). Endoscope with long axis gave us clear operative view but difficult to reach surgical instruments and intratumoral manipulation. Lower right: We could observe the surface of brainstem and basilar artery through dural defect. basilar artery (square), brainstem (circle).

A thirty-six-year-old male presented right motor weakness, diplopia, and facial sensory disturbance because of its oculomotor, trochlear, and trigeminal nerve impairment. CT and MRI showed enhanced mass lesion with calcification located clivus, and it extended toward brainstem and its lateral side. The first surgery was performed by craniotomy, and tumor was resected partially. Thirty months after surgery, residual tumor regrowth was detected. And a second transcranial surgery was performed. After the second surgery, cranial nerve palsy gradually improved. But fifty months after the second surgery, the tumor was growing toward brainstem and sphenoid sinus. The third surgery was performed by endoscopic endonasal transsphenoidal approach, because main part of tumor extension was midline. We performed the surgery with the same strategy as Case 1, that was using instruments that do not disturb the operative field without the interference by transphenoidal approach. We made suprasellar part of tumor remain intentionally, because suprasellar part was very hard, and we considered the possibility of the involvement of perforators and injury of hypothalamus. We could perform precise manipulation without intraoperative complications such as carotid artery and basilar artery, cranial nerves, and brainstem injuries. This case was also seen dural defect, and it was repaired by patched with muscle fascia at defect and filling sphenoid sinus by autologous fat and fibrin glue. After surgery, there were no new deficit and cerebrospinal fluid leakage. Immunohistochemically, epithelial membrane antigen (EMA) and cytokeratin (CAM5.2) were negative, and the pathological diagnosis was chondrosarcoma. Residual tumor regrowth was not observed for thirty months after surgery (Figures 3, 4 and 5).

## 3. Discussion

 Although there are various operative approaches for clival tumors [1–5], a transsphenoidal approach [6, 7] is one of choices when the main tumor extention is in an anterior-posterior direction with a slight lateral extension. 

Bouche et al. [8] reported transsphenoidal approach for clival tumor in 1966, but in those days, there was the limitation of removal of tumor which extend to lateral and deep part, because it is difficult to obtain sufficient microscopic operative view. As a result, this approach did not widely spread. 

 Recently, development of neuroendoscope gives us improvements of visibility of operative field. And innovation of surgical instruments that specialized in endoscopic transsphenoidal approach and image navigation systems gives us safe and precise surgical procedures [10]. 

 However, this approach sometimes provides only narrow and deep operative field. To remove the tumor which requires us to progress more into deeper and lateral parts, we have to use longer endoscope to observe details and instruments could be reached to those parts. Rhoton Jr. [11] described dissection instruments have shafts at least 120 mm in length when reach intrasuprasellar lesion for transspnenoidal surgery. When we consider the interference of bimanual manipulation and the surgical instruments inside and outside of the nasal cavity, we think that more longer endoscope and surgical instruments seems to be needed for clival lesion especially extend superior-posterior part. 

 In our case, we used endoscope with long axis (length: 180 mm) and angled tip (0, 30, and 70 degrees, HOPKINSII: KARL STORZ Co., Ltd.) that was able to observe deep operative field, superior and lateral part of lesion. And instruments which have long and slim curettes, ultrasonic aspirator probe for abdominal surgery, and high-speed drill which have curved handle were not to disturb operative field without the interference of each tool. These instruments gave us easy and precise manipulation of tumor removal. When we used neuronavigation system, we made our own long probe to navigate the deep part. And this information might make us avoid intraoperative complications of parasellar critical structures. 

 Cerebrospinal fluid leakage is a major complication of transsphenoidal surgery, and it is an important factor to get good results of this surgery. Frank et al. [12] reported that they experienced 18% CSF leakages after endoscopic transsphenoidal surgery. And CSF leakages were controlled by fat packing. To prevent this complication, we do reconstruction by fat packing, dural plasty using muscular fascia, pedicled nasoseptal flap, and fibrin glue. And these techniques were applied to parasellar lesion such as clival chondrosarcoma. 

 Almefty et al. [13] mentioned that chordoma and chondrosarcoma differed with regard to their origin and histology and differed markedly with regard to outcome. That is, chordoma type (chordoma and chondroid chordoma) demonstrated aggressive clinical course and poor outcome. On the other hand, chondrosarcoma patients had a significantly better outcome compared with chordoma patients with regard to survival and recurrence-free survival without high-dose radiotherapy. Almefty et al. mentioned that radiotherapy may not be necessary in patients with low-grade chondrosarcoma. Our cases were diagnosed low-grade chondrosarcoma, and their features were consistent with the concept of Almefty et al. group. 

 Recently, radiotherapy such as heavy ion irradiation develops as an additional treatment for clival tumors [9]. While less invasive surgical approach would be recommended, it might be one of a choice as a strategy for clival tumors, that is, a combination of subtotal resection and radiotherapy such as heavy ion irradiation for residual lesion to avoid complications of parasellar critical structures. 

 However, there are still insufficient measures for progression into deep and lateral parts, even if using our surgical instruments as the above. Especially, we need development of surgical instrument to reach the lateral part of the lesion. Further development and improvement of special instruments for transsphenoidal approach will be needed for clival tumors such as chondrosarcoma.

## Figures and Tables

**Figure 5:**